# Multilayer factors associated with excess all-cause mortality during the omicron and non-omicron waves of the COVID-19 pandemic: time series analysis in 29 countries

**DOI:** 10.1186/s12889-024-17803-8

**Published:** 2024-02-02

**Authors:** Fengjuan Zou, Jianpeng Xiao, Yingying Jin, Ronghua Jian, Yijun Hu, Xiaofeng Liang, Wenjun Ma, Sui Zhu

**Affiliations:** 1 Department of Epidemiology, School of Medicine, Jinan University, No.601 Huangpu Road West, Guangzhou, Guangdong 510632 China; 2 Guangdong Provincial Institute of Public Health, Guangdong Provincial Center for Disease Control and Prevention, Guangzhou, Guangdong 511430 China; 3 Disease Control and Prevention Institute, Jinan University, No.601 Huangpu Road West, Guangzhou, Guangdong 510632 China; 4 Chinese Preventive Medicine Association, Beijing, 100062 China

**Keywords:** COVID-19, The Omicron wave, Excess all-cause mortality, Influencing factors

## Abstract

**Background:**

The COVID-19 pandemic has resulted in significant excess mortality globally. However, the differences in excess mortality between the Omicron and non-Omicron waves, as well as the contribution of local epidemiological characteristics, population immunity, and social factors to excess mortality, remain poorly understood. This study aims to solve the above problems.

**Methods:**

Weekly all-cause death data and covariates from 29 countries for the period 2015–2022 were collected and used. The Bayesian Structured Time Series Model predicted expected weekly deaths, stratified by gender and age groups for the period 2020–2022. The quantile-based g-computation approach accounted for the effects of factors on the excess all-cause mortality rate. Sensitivity analyses were conducted using alternative Omicron proportion thresholds.

**Results:**

From the first week of 2021 to the 30th week of 2022, the estimated cumulative number of excess deaths due to COVID-19 globally was nearly 1.39 million. The estimated weekly excess all-cause mortality rate in the 29 countries was approximately 2.17 per 100,000 (95% CI: 1.47 to 2.86). Weekly all-cause excess mortality rates were significantly higher in both male and female groups and all age groups during the non-Omicron wave, except for those younger than 15 years (*P* < 0.001). Sensitivity analysis confirmed the stability of the results. Positive associations with all-cause excess mortality were found for the constituent ratio of non-Omicron in all variants, new cases per million, positive rate, cardiovascular death rate, people fully vaccinated per hundred, extreme poverty, hospital patients per million humans, people vaccinated per hundred, and stringency index. Conversely, other factors demonstrated negative associations with all-cause excess mortality from the first week of 2021 to the 30th week of 2022.

**Conclusion:**

Our findings indicate that the COVID-19 Omicron wave was associated with lower excess mortality compared to the non-Omicron wave. This study’s analysis of the factors influencing excess deaths suggests that effective strategies to mitigate all-cause mortality include improving economic conditions, promoting widespread vaccination, and enhancing overall population health. Implementing these measures could significantly reduce the burden of COVID-19, facilitate coexistence with the virus, and potentially contribute to its elimination.

**Supplementary Information:**

The online version contains supplementary material available at 10.1186/s12889-024-17803-8.

## Introduction

Globally, more than 640 million confirmed cases of coronavirus disease 2019 (COVID-19) and a staggering 6.6 million deaths were reported by the World Health Organization (WHO) as of December 2, 2022 [1]. Excess mortality refers to the number of deaths resulting from all causes during the COVID-19 pandemic that exceeds the expected number of deaths based on historical trends in all-cause mortality, which is a crucial measurement of the actual toll of the COVID-19 pandemic on the population [2, 3]. Estimations reveal that the global all-age rate of excess mortality during the period from January 1, 2020, to December 31, 2021, was 120.3 deaths per 100,000 individuals [2], placing an immense burden on healthcare, individuals, and the economy [4, 5]. Many public health interventions have been devised to mitigate the transmission of severe acute respiratory syndrome coronavirus 2 (SARS-CoV-2), including the implementation of wearing mask, widespread vaccination campaigns, and the practice of social distancing. However, the COVID-19 pandemic has undergone rapid evolution. The emergence of the Omicron (B.1.1.529) variant of SARS-CoV-2, initially identified in South Africa on November 26, 2021 [6], has been associated with potentially reduced severity but significantly heightened transmissibility compared to earlier variants [7], along with demonstrated immune evasion capabilities [8]. Consequently, this variant has triggered substantial Omicron outbreaks in numerous countries and has already had a profound global impact.

Many factors may account for the COVID-19 pandemic, including local epidemiology, population immunity, demographic composition, and healthcare system capacity. However, some important questions were raised: (1) Are there disparities in age- and sex-specific excess mortality between the Omicron and non-Omicron waves of COVID-19 worldwide during the period of 2021 and 2022? (2) To what extent can all-cause excess mortality be attributed to the local epidemiological characteristics of the population, SARS-CoV-2 variants, population immunity, and social determinants?

Sparse data have been documented regarding heterologous excess mortality concerning non-Omicron and Omicron variants in different countries. In this study, we conducted a comparative analysis of the surplus all-cause mortality between the non-Omicron and Omicron waves in 29 countries, examining the contributory factors associated with this phenomenon due to the pandemic, spanning from the initial week of 2021 to the 30th week of 2022.

## Methods

### Mortality data collection

We conducted a time series analysis using weekly mortality data collected from the Short-term Mortality Fluctuations data series of the Human Mortality Database (https://www.mortality.org/Data/STMF ) for the first week of 2015 to the 30th week of 2022. These data disaggregated by age and sex are provided for each country. These data are from the Max Planck Institute for Demographic Research in Rostock, Germany, as well as authoritative national agencies, and are supported by the Department of Demography at the University of California, Berkeley, USA [9].

The gender-specific national average population data for 29 countries were collected from the Human Mortality Database. Additionally, the population data for the 5-year age groups were sourced from the United Nations (https://population.un.org/wpp/Download/Standard/Population/).

### Variates of the COVID-19 virus collection

The COVID-19 virus variants, including the Alpha, Beta, Gamma, Delta, and Omicron variants, were collected biweekly from the Our World in Data database. This database is a collaborative effort between researchers at Oxford University and the nonprofit Global Change Data Lab (https://ourworldindata.org/organization). To address missing values for the variants during odd weeks, a duplicated method was employed where the value from the following week was used as a substitute. It is important to note that the current situation is characterized by the dominance of the Omicron variant, defined as having an Omicron proportion of 50% or higher.

### Covariate data collection

Three COVID-19 vaccine candidates, including the total boosters per hundred, people vaccinated per hundred, and people fully vaccinated per hundred, other covariates such as epidemic intensity of COVID-19 (including new confirmed cases of COVID-19 per million, positive rate, the effective reproduction rate of the virus, and new tests for COVID-19 per thousand), the capacity of the healthcare system (including COVID-19 patients in ICU per million, COVID-19 patients in hospital per million, hospital beds per thousand), the prevalence of chronic diseases (such as death rate from cardiovascular disease in 2017, diabetes prevalence of population aged 20 to 79 in 2017), and various social factors (including stringency index, population density, human development index, median age, aged ≥ 65 years old, aged ≥ 70 years old, the share of women or men who smoke, GDP, extreme poverty) were also collected from the Our World in Data database.

Most of these covariates were reported daily, except for newly confirmed cases of COVID-19 per million, COVID-19 patients in ICUs per million, and COVID-19 patients in hospitals per million. To facilitate future analysis, the weekly averages of these covariates were calculated.

### Statistics

All covariates were evaluated as non-normal continuous variables, and the median (P_50_), 25th percentile (P_25_), and 75th percentile (P_75_) were used to describe the distribution of nonnormally distributed data. Based on weekly all-cause death data from 29 countries for the period 2015–2019, before the COVID-19 pandemic, a Bayesian Structured Time Series Model (BSTS) was used to predict the expected weekly deaths and their 95% confidence intervals (95% CI) during the COVID-19 pandemic for the period 2020–2022. The predictions were stratified by gender and age groups, specifically 0–14, 15–64, 65–74, 75–84, and ≥ 85 years. The BSTS, a stochastic state space model, is employed to predict the expected number of deaths. It integrates trend, seasonality, and regression components, enabling a comprehensive time series data analysis [10]. The model automatically assimilates prior information and likelihood functions to derive a posterior distribution [11]. Estimating this distribution for each model parameter is meticulously carried out using the Markov Chain Monte Carlo (MCMC) algorithm. Subsequently, the Bayesian model averaging technique is applied, refining the results of the posterior distributions to achieve the final predictive output.

The BSTS model accounted for the exclusion of confounding effects arising from long-term trends and seasonal variations. The BSTS model consists of three main components: the Kalman filter, spike-and-slab method, and Bayesian model averaging [12], which enables it to consider time-varying model parameters at the same time and present the random characteristics of the target sequence [12, 13]. The prediction results of the BSTS method rarely depend on certain assumptions [13], which can effectively deal with the uncertainties contained in the time series [12, 14]. In the field of public health, the BSTS model is widely used in disease surveillance and prediction, causal inference, trend analysis, and policy evaluation [12, 15–17], and many previous studies have found that the BSTS model has better prediction performance than other time series models [13, 18]. The equations of BSTS are as follows:

1 $$ {y_t} = Z_t^T{\alpha _t} + {\mu _t} + {\tau _t} + {\varepsilon _{(t)}}{\varepsilon _t} \sim N(0,{H_t}) $$

2 $$ {\alpha }_{t+1}={T}_{t}{\alpha }_{t}+{R}_{t}{\eta }_{t} {\eta }_{t}\sim N\left(0,{Q}_{t}\right)$$

Equation 1 is the observation equation, while Eq. 2 is referred to as the state equation [16]. $$ {y}_{t}$$ is the expected number of all-cause deaths in week t. $$ {\alpha }_{t}$$ is the potential d-dimensional state vector for week t. $$ {Z}_{t}$$ is defined as the d-dimensional output vector. $$ {T}_{t}$$ referred to as the transition matrix, is a $$ d\times d$$ dimensional matrix that governs the evolution of the state vector$$ {\alpha }_{t} $$over time. $$ {R}_{t}$$ is identified as the $$ d\times q$$ control matrix, which, along with $$ {T}_{t}$$, contains a mix of known values (0 and 1). $$ {\epsilon }_{t }$$and $$ {\eta }_{t}$$ are Gaussian error terms with mean 0 and variances $$ {H}_{t}$$C and $$ {Q}_{t}$$. $$ {\mu }_{t}$$ and $$ {\tau }_{t}$$ are used to control for long-term trends and seasonal effects (53 weeks per year), respectively. The “CausalImpact” R package was used to estimate the expected number of deaths using a 1000-iteration MCMC algorithm.

We used meta-analysis to combine the weekly excess mortality and confidence interval of each country in 29 countries into the total effect value of each country in 29 countries and the total effect value of the world. Then, the all-cause excess mortality rate per 100,000 was computed and compared between the Omicron and non-Omicron waves of COVID-19. Wilcoxon’s rank sum tests were utilized for this comparison. Furthermore, subgroup analysis was performed, transforming the data by age and gender groups, to examine any variations within these categories.

To explore the impact and significance of all covariates on the excess all-cause mortality rate, we employed a two-step approach. First, Spearman’s Rank Correlation coefficient (*r*s) analysis was utilized to identify variables that showed a significant correlation (*P* < 0.05) with the all-cause excess mortality rate. This analysis helped to eliminate variables with weak or negligible relationships to the mortality rate. In the second step, a random forest (RF) regression model was used to assess the feature importance of explanatory variables in predicting the all-cause excess mortality rate. This approach aids in reducing the burden of data collection and enhances efficiency by identifying the most influential variables for making accurate predictions [19]. Furthermore, these covariables provided by the Our World in Data database have data missing, and some variables have overlapping meanings. In such cases, we prioritized variables that had greater significance in the RF model and fewer missing entries. The selected covariates underwent multiple imputation to address missing values, thereby ensuring their suitability for subsequent analysis. Next, to incorporate the impact of the mixture, the quantile-based g-comp.

utation approach, implemented in the R package “qgcomp” [20, 21], was employed. This approach considers various factors, such as vaccines, COVID-19 variants, the epidemic intensity of COVID-19, the capacity of the healthcare system, the prevalence of chronic diseases, and social factors, to analyse the all-cause excess mortality rate. Additionally, each covariate’s positive or negative correlations and weights on the all-cause mortality rate were evaluated. This method flexibly combines Weighted Quantile Sum Regression and the g-computation causal effect estimation method, exhibiting several characteristics inherent to the causal inference method [20].

3 $$ {{\text{Y}}_i} = {\beta _0} + \sum\limits_{{\text{j}} = 1}^{\text{c}} {{\beta _{\text{j}}}} {\text{X}}_{{\text{ji}}}^{\text{q}} + { \in _i}$$

${\text{Y}}_{i}$ is the all-cause excess mortality for observation *i*, ${\beta }_{0 }$is the intercept, ${\beta }_{\text{j}}$ is the effect size of exposure j, ${ \in _i}$ is the error term, ${\text{X}}_{\text{j}}^{\text{q}}$ is the quantized versions of ${\text{X}}_{\text{j}}$, and ${\text{X}}_{\text{j}}^{\text{q}}$= { 0,1,2,3}(${\text{X}}_{\text{j}}^{\text{q}}$=0,when the observed value of ${\text{X}}_{\text{j}}$ is less than the 25th quartile of this variable). If all exposures are directionally homogeneous in their association with outcomes, $\psi$is equal to the sum of the regression coefficients ($\psi= \sum\nolimits_{j = i}^c {{\beta _j}}$), representing the estimated combined effect of all exposures as a whole on outcomes, and ${w_k} = {\beta _k}/\sum\nolimits_j^c {{\beta _j}}$ is the weight for each exposure (indexed by *k*, and the weights are defined to sum to 1.0) [20]. In cases where direction homogeneity is not established, the quantile g-computation approach adjusts the weights by categorizing them as either negative or positive. These positive and negative weights represent the correlation direction between the exposure and outcome indicators.

Finally, we conducted sensitivity analyses using alternative Omicron thresholds (≥ 60% and ≥ 70%) and imputing the missing values for the variants during odd weeks using the mean values of the two weeks before and after. The analyses were carried out with R software (version 4.2.2). A two-sided *P* value of less than 0.05 represented statistical significance.

## Results

### Excess all-cause mortality during the first week of 2021 to the 30th week of 2022

**Fig. 1:**
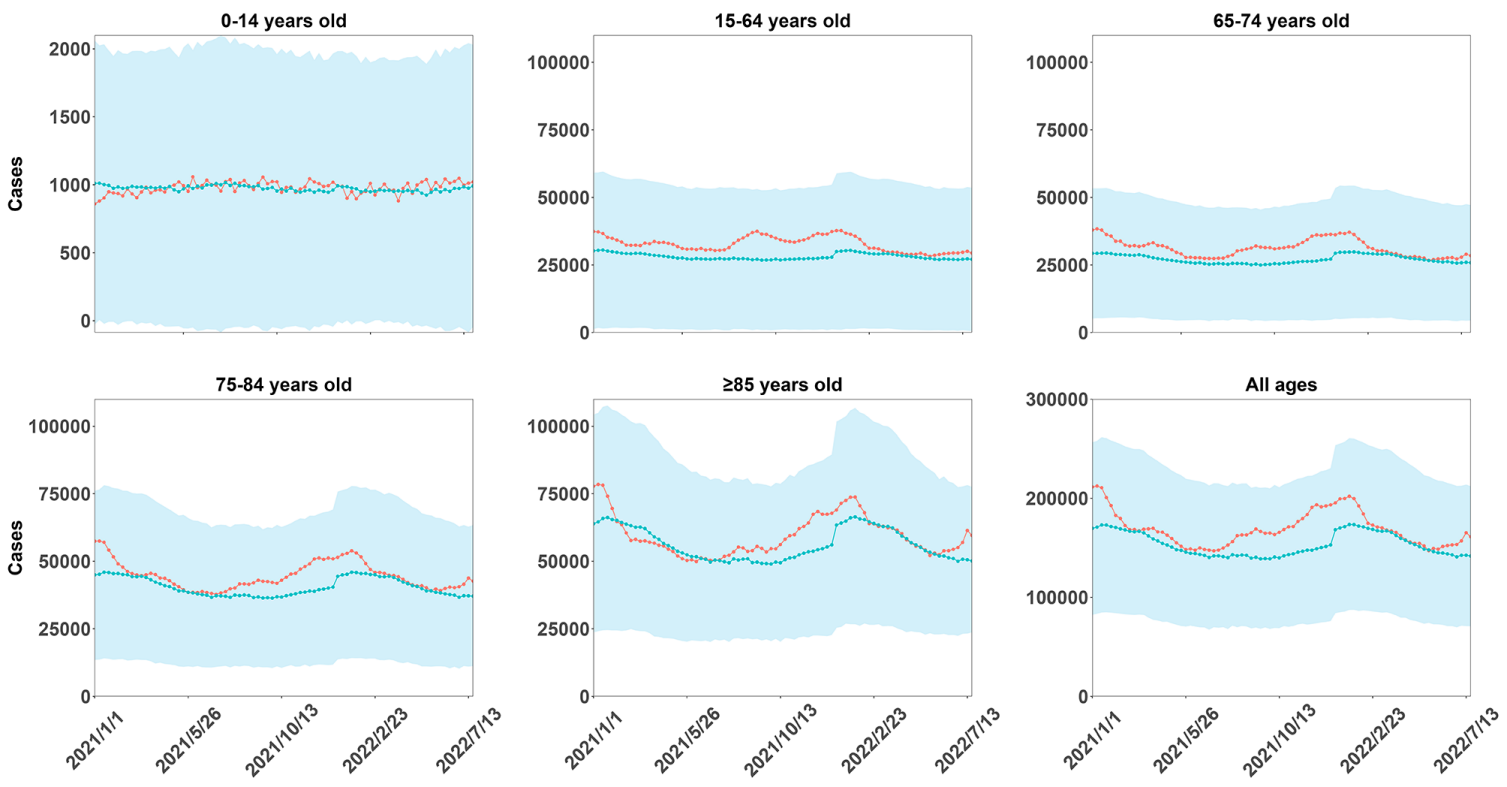
Age-specific weekly counts of observed and predicted deaths due to the COVID-19 pandemic globally. (The blue area means 95% confidence interval of the predicted)

The estimated cumulative number of excess deaths attributed to COVID-19 across all age groups globally was approximately 1.39 million, spanning from the first week of 2021 to the 30th week of 2022 (Table 1). Among the countries analysis, three countries (the United States, Poland, and Italy) witnessed cumulative excess deaths exceeding one hundred thousand during this period. In terms of age breakdown, the cumulative number of excess deaths for individuals under the age of 15 was nearly close to zero. However, the population aged ≥ 85 years experienced the highest cumulative number of excess deaths (Fig. 1). By the 30th week of 2022, the estimated weekly excess all-cause mortality rate was nearly 2.17 per 100,000 individuals (95% CI: 1.47 to 2.86) across the 29 countries/states. The magnitude of the all-cause excess mortality rate varied significantly among these countries. Bulgaria had the highest estimated weekly all-cause excess mortality rate, reaching 8.79 deaths (95% CI: 6.73 to 10.85) per 100,000 population. On the other hand, Luxembourg showed a negative all-cause excess mortality rate. In the majority of countries, the estimated excess all-cause mortality rate is higher among men than among women, with the exception of Spain, South Korea, Denmark, Luxembourg, and New Zealand. The weekly all-cause excess mortality rates due to the COVID-19 pandemic in 29 countries/states are shown in Figure S1.

**Table 1:** Number of reported deaths, excess deaths, and excess all-cause mortality rate weekly across 29 countries/states

Country/State	Reported deaths	Estimated deaths	Excess deaths	Excess mortality rate(per 100,000)	Excess mortality rate of Male(per 100,000)	Excess mortality rate of Female(per 100,000)
**Global**	13,873,219.00	12,539,884.33	1,333,334.67	2.17(1.47,2.86)	2.36(1.63,3.09)	1.91(1.25,2.57)
Israel	82,059.00	74,850.02	7,208.98	0.99(0.71,1.27)	1.22(0.89,1.54)	0.80(0.54,1.06)
South Korea	533,842.00	493,539.22	40,302.78	0.95(0.51,1.39)	0.92(0.52,1.32)	0.98(0.50,1.46)
Austria	141,948.00	131,925.24	10,022.76	1.39(0.97,1.81)	1.71(1.30,2.12)	1.04(0.58,1.50)
Belgium	179,337.00	174,481.34	4,855.66	0.49(0.09,0.90)	0.95(0.53,1.37)	0.09(-0.35,0.53)
Bulgaria	221,456.00	171,518.89	49,937.11	8.79(6.73,10.85)	9.42(7.23,11.61)	8.17(6.21,10.13)
Switzerland	112,749.00	108,077.53	4,671.47	0.65(0.26,1.04)	1.01(0.62,1.40)	0.28(-0.16,0.71)
Czechia	207,206.00	181,449.19	25,756.81	2.99(1.94,4.03)	3.54(2.29,4.79)	2.43(1.57,3.30)
Germany	1,611,217.00	1,535,320.82	75,896.18	1.12(0.58,1.65)	1.29(0.79,1.80)	0.92(0.36,1.49)
Denmark	91,357.00	88,643.38	2,713.62	0.56(0.18,0.93)	0.47(0.09,0.86)	0.65(0.24,1.06)
Spain	724,769.00	667,696.59	57,072.41	1.36(0.90,1.83)	1.52(1.09,1.94)	1.82(1.17,2.46)
Estonia	28,905.00	25,118.00	3,787.00	3.49(2.84,4.13)	3.89(3.17,4.62)	3.15(2.42,3.87)
Finland	93,386.00	88,028.22	5,357.78	1.18(0.82,1.53)	1.20(0.82,1.57)	1.17(0.77,1.56)
France	1,018,477.00	977,908.56	40,568.44	0.78(0.50,1.05)	1.13(0.86,1.40)	0.42(0.13,0.71)
Greece	228,656.00	201,186.23	27,469.77	3.21(2.47,3.95)	3.50(2.77,4.23)	2.91(2.13,3.70)
Croatia	96,832.00	81,401.76	15,430.24	4.61(3.58,5.63)	4.92(3.87,5.97)	4.31(3.26,5.36)
Italy	1,124,867.00	1,018,931.92	105,935.08	2.17(1.78,2.57)	2.29(1.92,2.67)	2.04(1.60,2.49)
Lithuania	72,259.00	58,354.63	13,904.37	6.04(5.09,6.99)	6.45(5.53,7.38)	5.70(4.64,6.76)
Luxembourg	7,062.00	7,283.34	-221.34	-0.42(-0.84,0.00)	-0.45(-0.99,0.08)	-0.35(-0.89,0.19)
Latvia	52,207.00	44,372.23	7,834.77	5.08(3.87,6.29)	5.31(4.16,6.47)	4.83(3.50,6.15)
Netherlands	262,853.00	250,272.80	12,580.20	0.88(0.40,1.37)	1.15(0.66,1.64)	0.63(0.13,1.13)
Norway	68,112.00	65,168.53	2,943.47	0.67(0.29,1.05)	0.75(0.36,1.13)	0.62(0.20,1.05)
Poland	781,820.00	672,316.89	109,503.11	3.48(2.50,4.47)	3.84(2.78,4.89)	3.14(2.20,4.09)
Portugal	198,568.00	174,210.67	24,357.33	2.90(1.81,3.99)	1.96(0.93,3.00)	1.64(0.70,2.58)
Slovakia	107,220.00	85,030.79	22,189.21	4.96(3.77,6.15)	5.16(3.90,6.42)	4.76(3.6,50.92)
Slovenia	36,099.00	33,927.94	2,171.06	1.26(0.61,1.90)	1.76(1.07,2.45)	0.71(0.02,1.40)
Sweden	140,567.00	136,996.76	3,570.24	0.40(0.12,0.69)	0.66(0.39,0.93)	0.15(-0.19,0.49)
United States	5,373,472.00	4,760,204.04	613,267.96	2.24(1.74,2.74)	2.36(1.80,2.92)	1.79(1.35,2.24)
New Zealand	57,252.00	54,812.63	2,439.37	0.57(0.31,0.84)	0.31(0.04,0.58)	0.76(0.45,1.06)
Chile	218,665.00	176,856.17	41,808.83	2.63(2.30,2.96)	2.88(2.51,3.25)	2.38(2.07,2.70)

### The excess all-cause mortality rate during omicron and non-omicron waves of COVID-19

**Fig. 2:**
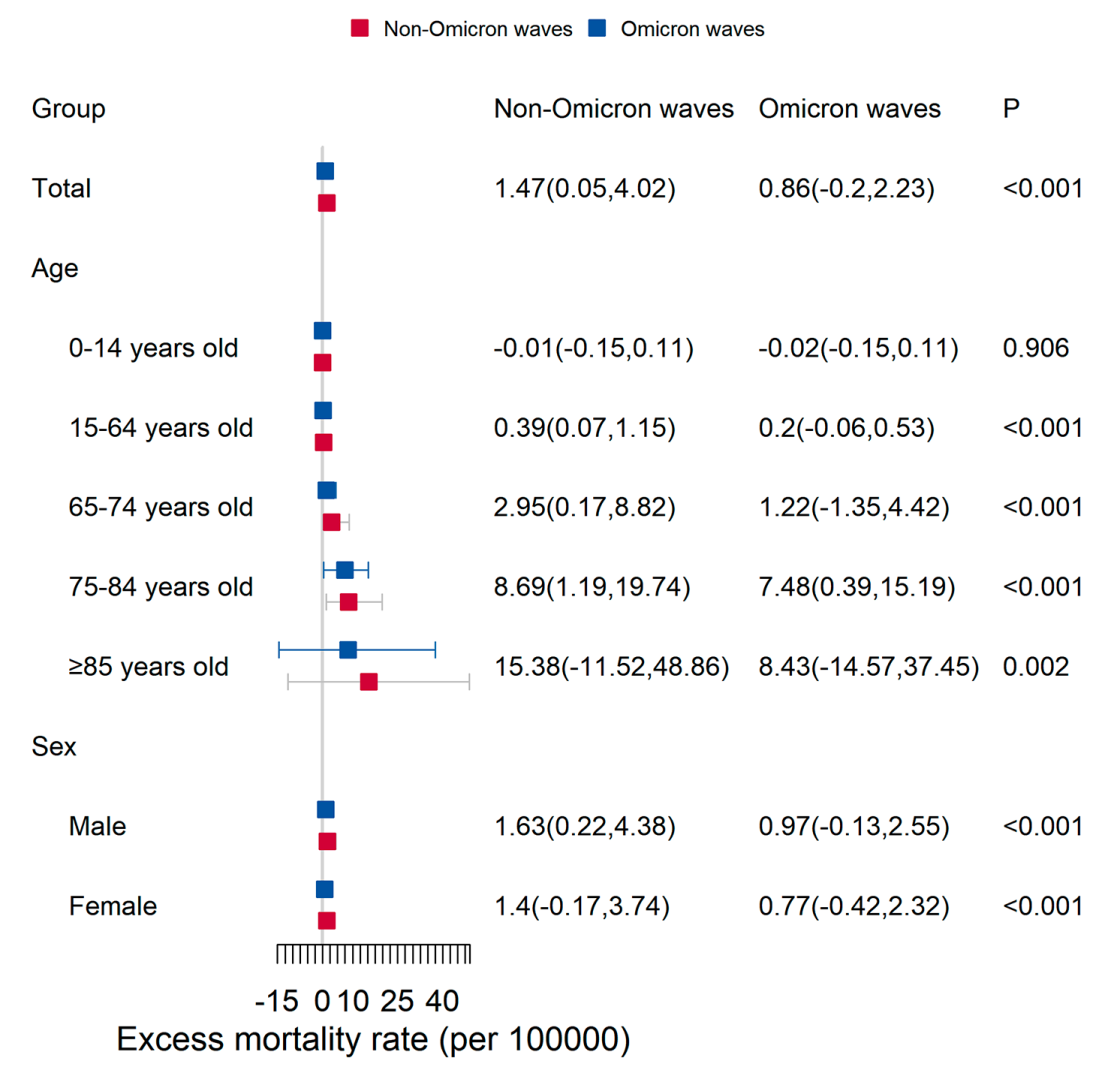
The weekly excess mortality rate during the Omicron and non-Omicron waves of COVID-19

Figure S2 (A) illustrates the weekly proportion of Omicron variants across the 29 countries/states during the study period. Starting in the 3rd week of 2022, the Omicron variant emerged as the predominant strain of COVID-19 worldwide. Additionally, the excess all-cause mortality rate during the Omicron wave of COVID-19 seemed to be lower than that during non-Omicron waves (Figure S2 B). Furthermore, across all age groups, the observed deaths were closely aligned with the expected levels during the COVID-19 Omicron wave (Fig. 1). During the non-Omicron wave, the estimated weekly excess all-cause mortality rate was 1.47 (0.05, 4.02) deaths per 100,000 population, whereas significantly less all-cause excess mortality was estimated during the COVID-19 Omicron wave, with 0.86 (-0.20, 2.23) deaths per 100,000 population in 29 countries/states globally (Fig. 2, *P* < 0.001). Comparatively, during the Omicron wave, the weekly all-cause excess mortality rate was lower in most countries, except for Belgium, Chile, Finland, New Zealand, Norway, South Korea, Spain, and the United States (Table S1). Figure 2 displays the age- and sex-stratified weekly all-cause excess mortality rates during the study period across 29 countries/states. Among individuals below the age of 15 years, there was no significant difference in all-cause excess mortality rates (*P = 0.906*), with the rate being close to zero. In comparison, the other four age groups exhibited lower weekly all-cause excess mortality rates during the COVID-19 Omicron wave when compared to the non-Omicron waves (*P* < 0.001). Interestingly, individuals in the age group of 85 years or older experienced the highest all-cause excess mortality rate in both the COVID-19 Omicron and non-Omicron waves. Then, the estimated all-cause excess mortality rate during the non-Omicron wave was approximately twice as high as that during the Omicron wave for both males and females.

### Characteristics of covariates

By considering the correlation of covariates, the contribution from the RF, and accounting for missing values, a total of 19 covariates were ultimately included (Figure S3). Table 2 presents the characteristics of these covariates during the Omicron and non-Omicron waves. During the study period, an median of 4500 new confirmed COVID-19 cases per million were reported weekly across the 29 countries/states, with the Omicron wave experiencing nearly three times the number of cases compared to the non-Omicron wave.

**Table 2:** The summary characteristics of included covariates in the Omicron and Non-Omicron waves across 29 countries/states

Covariates	Omicron waves				Non-Omicron wavse		
	Median	P_25_	P_75_		Median	P_25_	P_75_
People vaccinated per hundred	76.05	69.61	81.40		44.49	11.99	65.24
People fully vaccinated per hundred	71.84	64.98	78.48		29.65	4.46	58.44
Total boosters per hundred	51.53	33.68	58.50		0.00	0.00	0.43
New cases per million	4,500.12	1,419.59	11,097.22		1,156.28	416.81	2,368.57
Reproduction rate	1.00	0.79	1.24		1.03	0.87	1.20
ICU patients per million	39.37	3.77	103.83		70.61	12.39	241.48
Hospital patients per million	541.32	25.52	1,317.99		491.85	108.25	1,488.19
Test per case	4.87	3.06	9.37		21.97	10.65	60.94
Stringency index	24.07	14.81	37.68		51.14	41.25	65.51
Aged 70 older	12.93	10.20	13.75		12.93	10.20	13.75
Female smokers	23.10	19.30	28.20		23.10	19.30	28.20
GDP per capita	35,220.08	29,481.25	45,436.69		35,220.08	29,481.25	45,436.69
Extreme poverty	0.70	0.20	1.20		0.70	0.50	1.00
Cardiovascular death rate	133.98	114.32	227.33		133.98	114.32	227.33
Diabetes prevalence	5.81	4.79	7.17		5.81	4.79	7.17
Omicron	1.00	1.00	1.00		0.00	0.00	0.00
Non-Omicron	0.00	0.00	0.00		1.00	1.00	1.00
Positive rate	0.20	0.10	0.32		0.05	0.02	0.09
Human development index	0.92	0.88	0.93		0.92	0.88	0.93

### Contribution of covariates to excess all-cause mortality

**Fig. 3:**
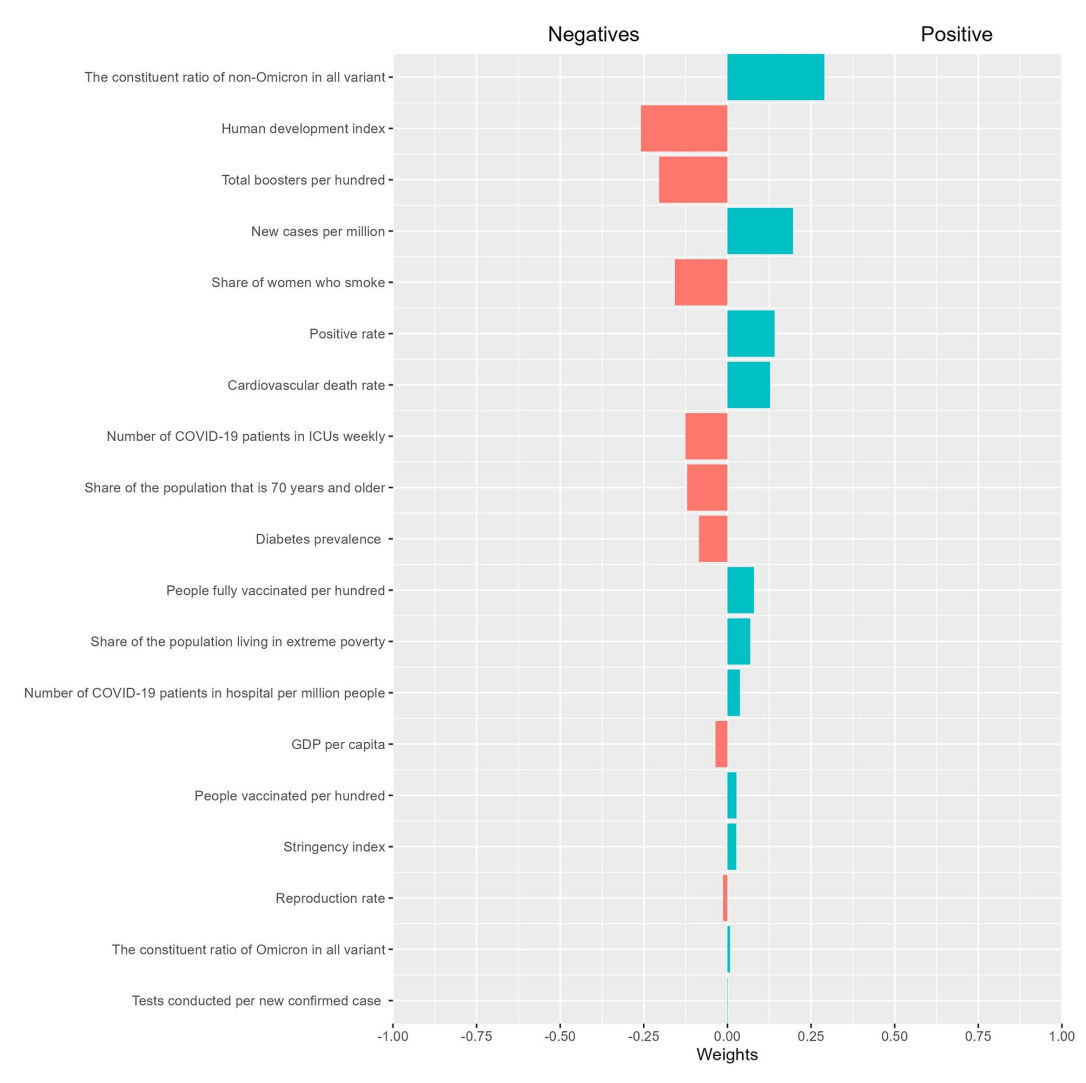
The “weights” of influencing factors correspond to the excess all-cause mortality

Figure 3 shows the findings of the joint effects analysis based on the qgcomp method. The results demonstrate that the proportion of non-Omicron variants in all variants, new cases per million, positive rate, cardiovascular death rate, people fully vaccinated per hundred, extreme poverty, hospital patients per million humans, people vaccinated per hundred, and stringency index exhibited a positive association with all-cause excess mortality rates. On the other hand, other factors displayed a negative relationship with all-cause excess mortality rates. Among the factors negatively associated with all-cause excess mortality rates, the human development index, the total number of COVID-19 vaccination boosters administered per 100 people, and female smokers accounted for the top three weights, collectively contributing to 62% of the overall weight. Conversely, among the factors positively associated with all-cause excess mortality rates, the constituent ratio of non-Omicron variants in all variants, the new confirmed cases of COVID-19 per million per week, and the positive rate held the most substantial weights, contributing to a total weight of 62.64%.

### Sensitivity analysis

In sensitivity analyses using different Omicron proportion thresholds (≥ 60% and ≥ 70%), the findings were consistent with those of the main analysis. Specifically, we observed that the weekly all-cause excess mortality rates during the non-Omicron waves of COVID-19 were significantly higher than those during the Omicron wave. This trend was evident in both male and female groups and across all age groups, except for individuals below the age of 15 years (Table S2). Furthermore, we noted slight variations in the all-cause excess mortality rates between the Omicron and non-Omicron waves when different criteria were used to define the Omicron wave (refer to Table S2). After re-imputing the data using the average of the two weeks before and after the missing data point, the results demonstrated that the revised imputation method did not significantly alter the findings (Table S3).

## Discussion

The comprehension of the impact of all-cause excess mortality during the non-Omicron and Omicron waves of the COVID-19 pandemic, as well as the factors contributing to it, holds significant importance for informing public health decision-making. Our study represents the first comprehensive global analysis of the factors influencing all-cause excess mortality during the COVID-19 epidemic, considering socioeconomic factors, vaccination rates, COVID-19 epidemic intensity, chronic disease prevalence, and healthcare system capacity. This research provides valuable insights into both the direct and indirect consequences of the COVID-19 pandemic on all-cause mortality.

Our analysis reveals that during the non-Omicron wave, the estimated all-cause excess mortality rate globally was 1.47 deaths per 100,000 population, which is about 1.7 times higher than that during the Omicron wave. Our findings suggest that several factors contribute to the burden of all-cause excess mortality. These include the constituent ratio of non-Omicron variants, the epidemic intensity of COVID-19, the percentage of people vaccinated with one or two doses, the stringency index, extreme poverty, cardiovascular death rate, and the number of hospital patients per million.

According to our findings, the all-cause excess mortality during the Omicron period was significantly lower than that during the non-Omicron period. This could be attributed to certain characteristics of the Omicron variant. For instance, spike mutations in the Omicron variant might impede the virus’s ability to utilize TMPRSS2, which is crucial for viral entry and replication in cells such as Calu3 and Caco2 cells. As a result, the replication capacity of the Omicron variant is substantially reduced both in vitro and in vivo when compared to the non-Omicron variants [22].

In addition, a study showed that compared with hamsters infected with non-Omicron variants, hamsters infected with Omicron variants had a significantly lower index of bronchiolitis, alveolitis, type II pneumocytosis and large type II pneumocytosis, and the spread of the virus in the lung tissue was reduced so that the Omicron variant showed a lower pathogenicity [23]. These findings suggest that the Omicron variant is associated with decreased severity of lung tissue infection and pathology in the hamster model.

Based on the observed properties of reduced pathogenicity and lower replication rate of the Omicron variant, we propose that the all-cause excess mortality attributed to COVID-19 is lower during the Omicron period than during the non-Omicron period. Our proposed hypothesis aligns with the findings of previous studies, which have also reported decreased mortality rates and lower severity of infection associated with the Omicron variant [24, 25]. The reduced mortality due to COVID-19 may be one of the reasons why excess all-cause mortality is lower in Omicron waves than in non-Omicron waves. Similar to our study, the excess all-cause death rate during the Omicron waves is also reportedly lower than it was during the non-Omicron period according to numerous studies [26]. However, a study by Massachusetts noted that the excess all-cause mortality rate during the omicron period was higher than that during the Delta period [27]. In this study, the observation period for the Omicron variant was only 8 weeks, from December 2021 to February 2022, which coincided with the winter season. The contradiction between this study and our findings can be attributed to the short observation period and the fact that it was conducted during a time when viral and cardiovascular disorders were more prevalent.

After employing the qgcomp method to examine the factors influencing all-cause excess mortality, we identified several key findings. The constituent ratio of non-Omicron in all variants, the intensity of the COVID-19 epidemic, the percentage of people vaccinated with only one or two doses per hundred, the stringency index, extreme poverty, cardiovascular death rate, and the number of hospital patients per million humans were identified as risk factors for all-cause excess mortality. Conversely, the socioeconomic indicator of the human development index, the administration of COVID-19 booster vaccines per 100 people, the proportion of the population aged 70 years and older, the percentage of women who smoke and the number of COVID-19 patients in intensive care units (ICU) were identified as protective factors. The strain on the healthcare system during the COVID-19 period may have led to individuals who required medical treatment being unable to access it [28, 29]. Furthermore, studies have indicated that infection with the virus responsible for COVID-19 can result in a decline in immune function [30], the prevalence of COVID-19 can indirectly impact people’s physical and mental health. Moreover, the widespread presence of COVID-19 can lead to various challenges in people’s lives and economies, which may result in a higher prevalence of mental or physical illnesses and subsequently increase the risk of death. In short, a higher number of new cases and a higher positive rate of COVID-19 are associated with increased deaths caused by COVID-19.

Research conducted by Akram Hernández-Vásquez indicates that individuals with a low human development index in Metropolitan Lima experienced higher rates of all-cause excess mortality during the COVID-19 pandemic [16], which aligns with our findings. Additionally, studies have demonstrated that the emergence of the Omicron variant is associated with a weakened neutralizing antibody response after receiving two vaccination doses. However, administering a third dose or boosters has been shown to generate a broader and higher antibody titer, which may help address the issue of reduced neutralization against the Omicron variant. These findings support the potential effectiveness of additional vaccine doses in mitigating the impact of the Omicron variant [31–33].

Contrary to prevalent assumptions, an inverse correlation was observed between the proportion of the population aged 70 years and older, the percentage of women who smoke and the number of COVID-19 patients in ICU, with the all-cause excess mortality. This finding appeared paradoxical at first but gained plausibility upon broader examination. Typically, individuals such as the elderly, smokers, and critically ill COVID-19 patients, who were more susceptible to adverse health outcomes, would exhibit higher mortality rates from infectious diseases. However, the protective measures implemented during the COVID-19 pandemic, such as restrictions and lockdowns, inadvertently shielded these vulnerable groups, potentially decreasing their mortality rates under these unique circumstances [34]. This pattern has been documented in various studies focusing on COVID-19 and environmental health, particularly during events like severe air pollution, heatwaves, cold spells, and epidemics [35]. It has been conceptualized as the ‘dry tinder’ effect [36], the ‘reverse harvesting effect’, and the ‘harvesting effect’ in different studies [35, 37–39].

To elucidate, consider the case of the elderly, who generally engage in fewer outdoor activities due to retirement or physical limitations, a phenomenon extensively studied in medical research [40–42]. The COVID-19 pandemic, with its community lockdowns, social distancing mandates, and heightened self-preservation instincts among the elderly, further curtailed their outdoor activities [43]. Consequently, this could lead to a reduced incidence of COVID-19 infection and accidental injuries among the elderly, potentially explaining the observed lower all-cause excess mortality rates in this demographic. Additionally, an increase in COVID-19 patients in ICUs might prompt hospitals to allocate more resources, equipment, and specialized care, thereby enhancing patient treatment and monitoring. This increased support could mitigate the risk of premature mortality due to resource constraints, offering a plausible explanation for the lower excess mortality rates observed with a higher number of ICU patients.

However, this article also has many limitations that cannot be ignored. First, since the population figures for each country in 2022 were unavailable at the start of the study, the average population for each country in 2020 and 2021 was used throughout the study period. However, internal migration or displacement may have occurred, leading to a potential underestimation of the number of excess deaths [44]. Furthermore, the study was limited by the availability and integrity of data. Only 29 countries were included in the analysis, and the study did not encompass a global analysis of excess deaths in each country. As a result, it is important to exercise caution when generalizing the conclusions of this study to every country worldwide. These limitations highlight the need for ongoing research and data collection to improve our understanding of all-cause excess mortality and its factors on a global scale.

## Conclusion

In conclusion, our study findings demonstrate that the all-cause excess mortality during the Omicron waves of COVID-19 was lower than that during the non-Omicron waves. Factors such as a high proportion of non-Omicron variants, increased new cases, and a high positive rate were identified as risk factors for all-cause excess mortality. Conversely, a high human development index, administration of a third dose of vaccination, and a population over 70 years old were identified as protective factors. Furthermore, extreme poverty, cardiovascular death rate, and hospital patients per million humans were found to be risk factors for all-cause excess mortality, while booster vaccinations were identified as protective factors. It is recommended to focus on improving the economy, promoting booster vaccinations, and enhancing overall population health to effectively reduce all-cause excess mortality. These efforts will contribute to achieving the goal of harmonious coexistence with COVID-19 and potentially even eliminating the virus from humanity.

### Electronic supplementary material

Below is the link to the electronic supplementary material.

Supplementary Material 1

## Data Availability

This research has been conducted using public resources: the Human Mortality Database, the Human Mortality Database, and the United Nations. The data presented in this study are available on reasonable request from the corresponding author.

## Abbreviations

COVID-19: Coronavirus disease 2019
SARS-CoV-2: Severe acute respiratory syndrome coronavirus 2
BSTS: Bayesian Structured Time Series Model
RF: Random forest
qgcomp: The quantile-based g-computation
rs: Spearman’s Rank Correlation coefficient
ICU: intensive care units
