# Supplementary material for: Multilayer factors associated with excess all-cause mortality during the omicron and non-omicron waves of the COVID-19 pandemic: time series analysis in 29 countries
Source: BMC Public Health. 2024 Feb 2;24:350. doi: 10.1186/s12889-024-17803-8 (PMC10835930; doi:10.1186/s12889-024-17803-8)
Supplement: Supplementary file 1 — Supplementary Material 1 [file 12889_2024_17803_MOESM1_ESM.docx]

***Supplementary materials***

**Multilayer factors associated with excess all-cause mortality during the Omicron and non-Omicron waves of the COVID-19 pandemic: time series analysis in 29 countries**


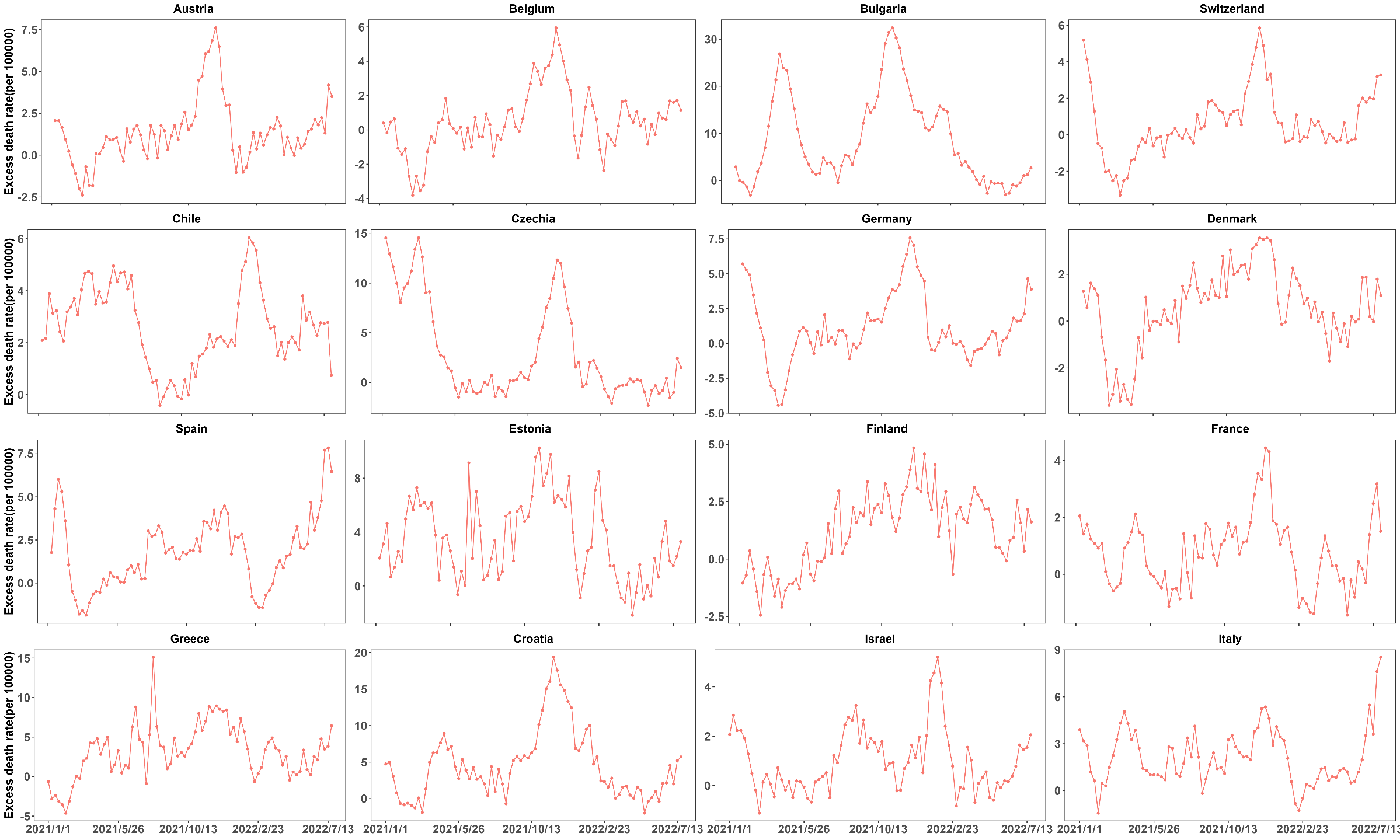


**Figure S1.** The weekly excess mortality due to the COVID-19 pandemic across 29 countries/states.

**
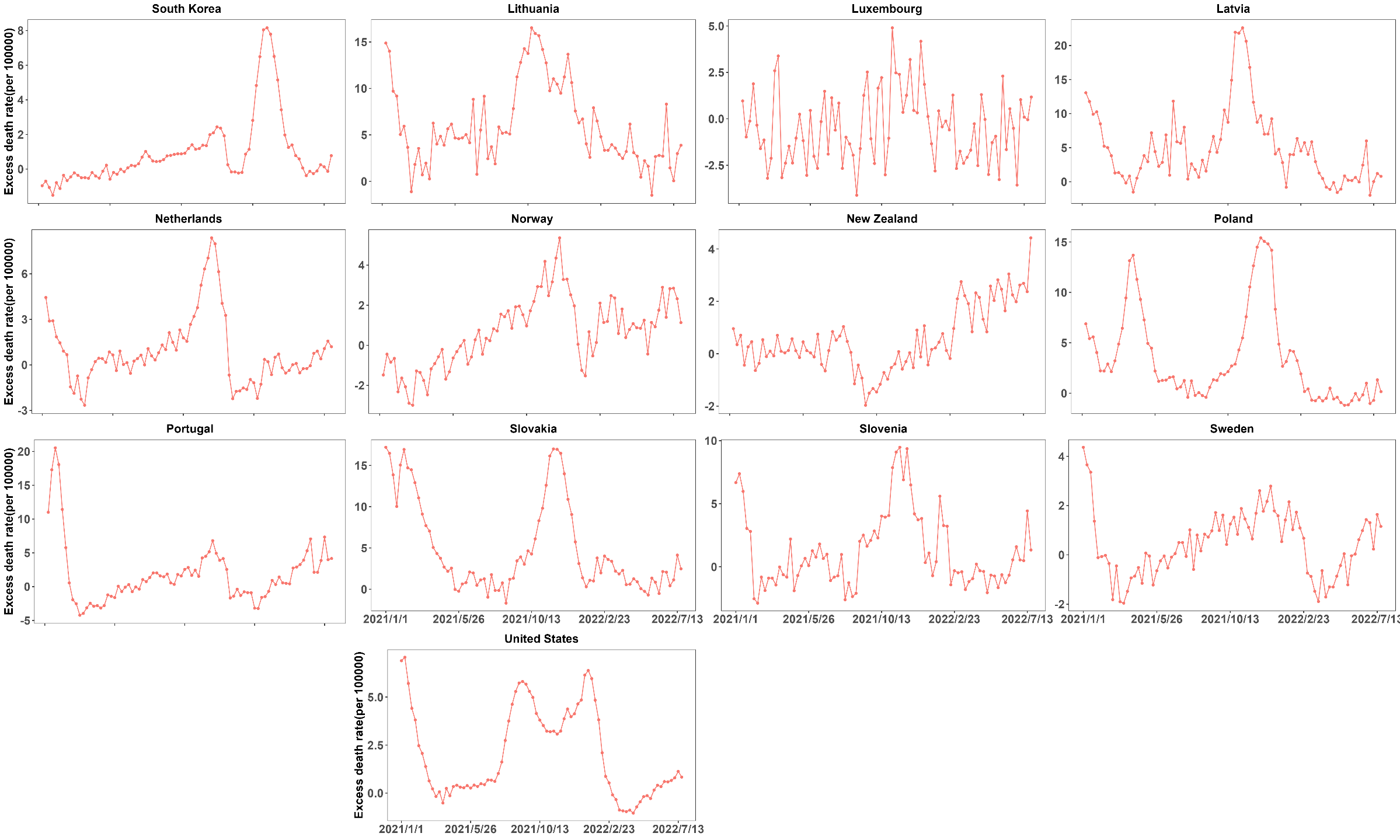
**

**Figure S1**. The weekly excess mortality due to the COVID-19 pandemic across 29 countries/states (Continued).


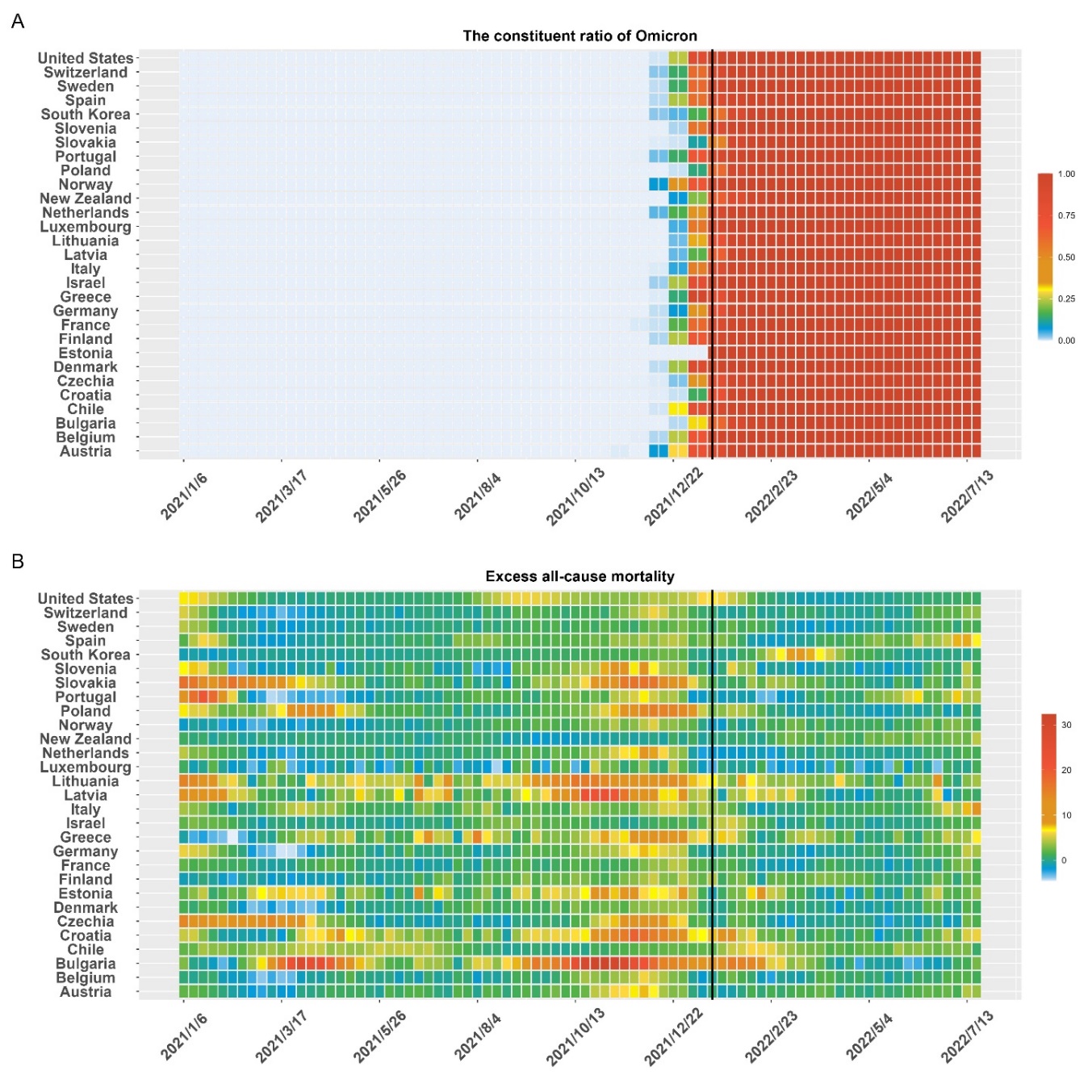


**Figure S2.** The constituent ratio of Omicron (A) and weekly excess all-cause mortality rate (B) across 29 countries/states. (Black vertical line indicates the third week of 2022)

**
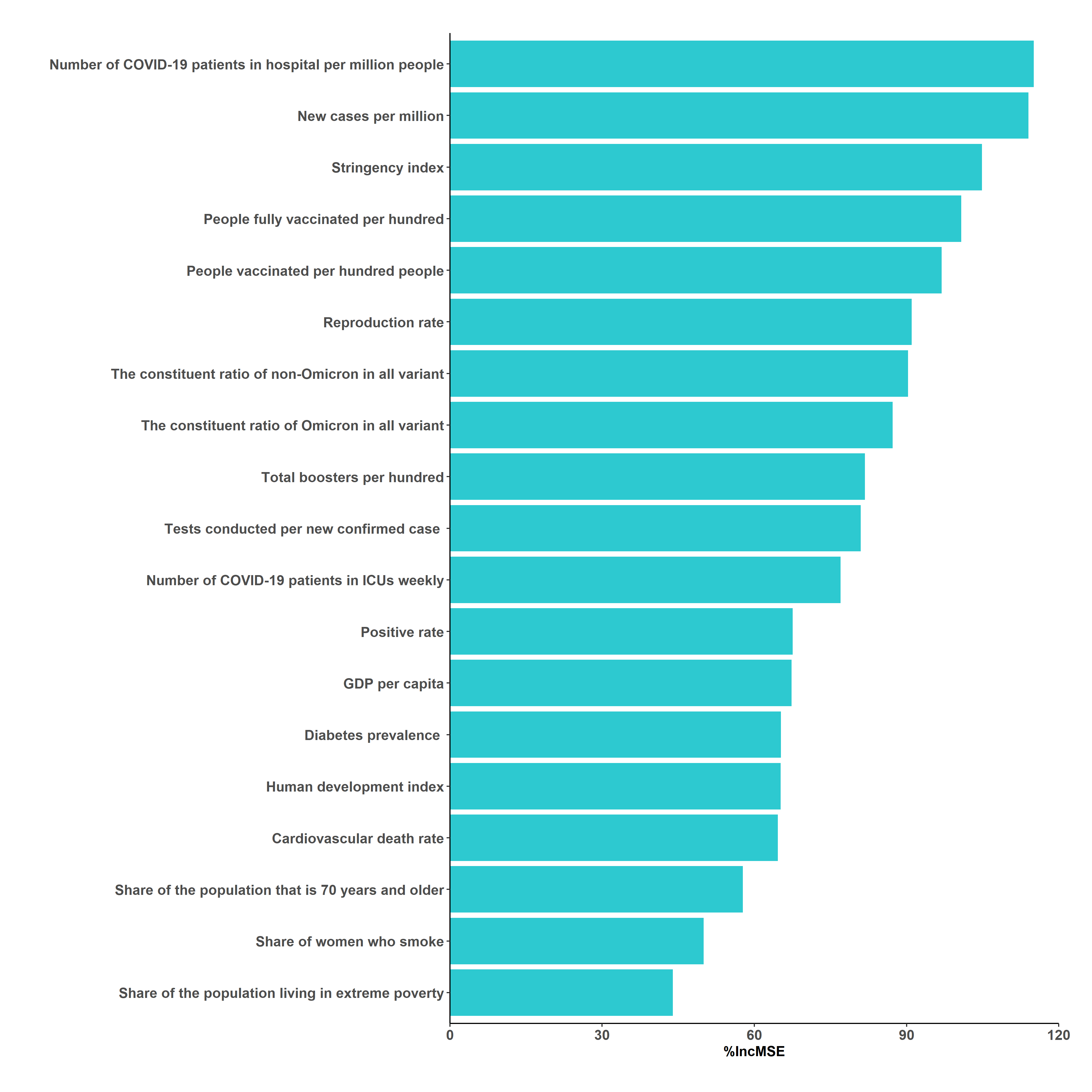
**

**Figure S3.** Ranking of contribution variables to excess mortality rate due to the COVID-19 pandemic.

**Table S1** The weekly excess deaths and mortality rate by Country for Omicron and Non-Omicron Periods.

| Location | variates | Excess deaths | The weekly excess mortality rate (per 100 000) | *Z* | *P* |
| --- | --- | --- | --- | --- | --- |
| Austria | Omicron | 101.34(33.69,144.98) | 1.14(0.38,1.63) | 42.21 | 0.479 |
|  | Non-Omicron | 109.6(25.13,183.35) | 1.23(0.28,2.06) |  |  |
| Belgium | Omicron | 69.89(-35.3,149.05) | 0.60(-0.30,1.29) | 42.21 | 0.740 |
|  | Non-Omicron | 28.53(-69.13,204.67) | 0.25(-0.60,1.77) |  |  |
| Bulgaria | Omicron | 78.33(-45.68,385.62) | 1.13(-0.66,5.56) | 42.20 | <0.001 |
|  | Non-Omicron | 745.78(232.50,1245.66) | 10.76(3.35,17.97) |  |  |
| Chile | Omicron | 525.08(389.63,698.29) | 2.71(2.01,3.60) | 42.22 | 0.234 |
|  | Non-Omicron | 441.61(222.91,727.40) | 2.28(1.15,3.75) |  |  |
| Croatia | Omicron | 76.46(20.91,188.04) | 1.87(0.51,4.61) | 42.21 | 0.008 |
|  | Non-Omicron | 203.65(88.97,281.40) | 4.99(2.18,6.90) |  |  |
| Czechia | Omicron | -31.98(-88.79,40.18) | -0.30(-0.84,0.38) | 42.20 | <0.001 |
|  | Non-Omicron | 241.59(19.21,991.07) | 2.30(0.18,9.42) |  |  |
| Denmark | Omicron | 16.49(-2.79,64.24) | 0.28(-0.05,1.10) | 42.21 | 0.373 |
|  | Non-Omicron | 59.13(-27.58,107.38) | 1.01(-0.47,1.84) |  |  |
| Estonia | Omicron | 19.93(-1.25,39.68) | 1.50(-0.09,2.99) | 42.20 | <0.001 |
|  | Non-Omicron | 62.57(25.37,82.52) | 4.71(1.91,6.21) |  |  |
| Finland | Omicron | 103.06(52.56,130.39) | 1.86(0.95,2.36) | 42.22 | 0.027 |
|  | Non-Omicron | 37.72(-39.08,123.21) | 0.68(-0.71,2.23) |  |  |
| France | Omicron | 191.46(-204.91,895.33) | 0.30(-0.32,1.39) | 42.21 | 0.086 |
|  | Non-Omicron | 700.62(27.60,980.56) | 1.09(0.04,1.52) |  |  |
| Germany | Omicron | 216.19(-214.48,872.52) | 0.26(-0.26,1.05) | 42.21 | 0.117 |
|  | Non-Omicron | 768.99(-29.79,2859.02) | 0.92(-0.04,3.43) |  |  |
| Greece | Omicron | 348.43(97.34,463.27) | 3.33(0.93,4.42) | 42.21 | 0.479 |
|  | Non-Omicron | 384.43(95.48,597.40) | 3.67(0.91,5.70) |  |  |
| Israel | Omicron | 59.24(8.62,145.04) | 0.67(0.10,1.64) | 42.21 | 0.988 |
|  | Non-Omicron | 71.54(12.56,154.91) | 0.81(0.14,1.75) |  |  |
| Italy | Omicron | 744.80(348.98,1857.59) | 1.25(0.59,3.13) | 42.21 | 0.057 |
|  | Non-Omicron | 1294.37(649.43,1956.60) | 2.18(1.09,3.30) |  |  |
| Latvia | Omicron | 15.70(-0.94,75.16) | 0.83(-0.05,3.99) | 42.20 | <0.001 |
|  | Non-Omicron | 102.60(44.42,180.86) | 5.44(2.36,9.59) |  |  |
| Lithuania | Omicron | 85.14(71.53,111.15) | 3.04(2.55,3.96) | 42.20 | <0.001 |
|  | Non-Omicron | 169.47(119.3,306.51) | 6.05(4.26,10.93) |  |  |
| Luxembourg | Omicron | -3.49(-12.61,0.73) | -0.55(-1.99,0.12) | 42.21 | 0.238 |
|  | Non-Omicron | -3.00(-12.14,8.38) | -0.47(-1.91,1.32) |  |  |
| Netherlands | Omicron | -36.56(-176.88,74.38) | -0.21(-1.01,0.43) | 42.20 | <0.001 |
|  | Non-Omicron | 154.36(27.31,449.83) | 0.88(0.16,2.58) |  |  |
| New Zealand | Omicron | 105.21(42.78,127.19) | 2.06(0.84,2.50) | 42.23 | <0.001 |
|  | Non-Omicron | 1.78(-25.72,23.43) | 0.03(-0.50,0.46) |  |  |
| Norway | Omicron | 61.28(33.03,104.08) | 1.14(0.61,1.93) | 42.22 | 0.049 |
|  | Non-Omicron | 12.79(-54.07,95.76) | 0.24(-1.00,1.78) |  |  |
| Poland | Omicron | -109.94(-273.53,410.82) | -0.29(-0.71,1.07) | 42.20 | <0.001 |
|  | Non-Omicron | 1170.84(502.72,2877.29) | 3.05(1.31,7.50) |  |  |
| Portugal | Omicron | 54.35(-91.95,326.97) | 0.53(-0.89,3.18) | 42.21 | 0.769 |
|  | Non-Omicron | 145.44(-88.86,319.67) | 1.41(-0.86,3.11) |  |  |
| Slovakia | Omicron | 71.18(32.31,120.82) | 1.31(0.59,2.22) | 42.20 | <0.001 |
|  | Non-Omicron | 244.69(78.30,666.14) | 4.49(1.44,12.22) |  |  |
| Slovenia | Omicron | -7.41(-15.57,11.64) | -0.35(-0.74,0.55) | 42.21 | 0.073 |
|  | Non-Omicron | 23.90(-17.54,81.65) | 1.13(-0.83,3.85) |  |  |
| South Korea | Omicron | 426.98(-58.23,1958.41) | 0.82(-0.11,3.78) | 42.22 | 0.004 |
|  | Non-Omicron | 114.95(-202.17,459.36) | 0.22(-0.39,0.89) |  |  |
| Spain | Omicron | 939.74(396.22,1422.95) | 1.98(0.84,3.00) | 42.22 | 0.479 |
|  | Non-Omicron | 811.68(110.44,1436.12) | 1.71(0.23,3.03) |  |  |
| Sweden | Omicron | 40.24(-86.69,132.30) | 0.39(-0.83,1.27) | 42.21 | 0.554 |
|  | Non-Omicron | 48.20(-38.70,144.82) | 0.46(-0.37,1.39) |  |  |
| Switzerland | Omicron | 30.31(-23.65,103.87) | 0.35(-0.27,1.20) | 42.21 | 0.806 |
|  | Non-Omicron | 29.50(-44.45,144.92) | 0.34(-0.51,1.67) |  |  |
| The United States | Omicron | 1593.76(-1074.79,3585.08) | 0.47(-0.32,1.07) | 42.20 | 0.004 |
|  | Non-Omicron | 9781.74(1400.47,14139.83) | 2.91(0.42,4.20) |  |  |
| Total | Omicron | 66.02(-11.82,218.69) | 0.86(-0.20,2.23) | 19.73 | <0.001 |
|  | Non-Omicron | 122.83(2.03,507.41) | 1.47(0.05,4.02) |  |  |

**Table S2.** The Wilcoxon test for weekly excess mortality with different Omicron waves definition criteria.

|  | Variants |  | Omicron proportion ≥60% | | | | |  | Omicron proportion ≥70% | | | | |
| --- | --- | --- | --- | --- | --- | --- | --- | --- | --- | --- | --- | --- | --- |
|  |  |  | Median | P_25_ | P_75_ | *Z* | *P* |  | Median | P_25_ | P_75_ | *Z* | *P* |
| All | Omicron |  | 0.86 | -0.21 | 2.23 | 19.91 | <0.001 |  | 0.85 | -0.22 | 2.23 | 20.17 | <0.001 |
|  | Non-Omicron |  | 1.46 | 0.05 | 4.02 |  |  |  | 1.46 | 0.05 | 3.97 |  |  |
| 0-14 years | Omicron |  | -0.02 | -0.15 | 0.11 | 23.05 | 0.926 |  | -0.02 | -0.15 | 0.11 | 23.48 | 0.633 |
|  | Non-Omicron |  | -0.01 | -0.15 | 0.11 |  |  |  | -0.02 | -0.15 | 0.11 |  |  |
| 15-64 years old | Omicron |  | 0.20 | -0.05 | 0.52 | 18.46 | <0.001 |  | 0.20 | -0.05 | 0.53 | 18.84 | <0.001 |
|  | Non-Omicron |  | 0.39 | 0.06 | 1.14 |  |  |  | 0.39 | 0.06 | 1.13 |  |  |
| 65-74 years old | Omicron |  | 1.17 | -1.36 | 4.42 | 18.09 | <0.001 |  | 1.22 | -1.43 | 4.43 | 18.42 | <0.001 |
|  | Non-Omicron |  | 2.96 | 0.19 | 8.73 |  |  |  | 2.94 | 0.18 | 8.67 |  |  |
| 75-84 years old | Omicron |  | 7.46 | 0.42 | 15.19 | 21.18 | <0.001 |  | 7.30 | 0.36 | 15.11 | 21.25 | <0.001 |
|  | Non-Omicron |  | 8.67 | 1.17 | 19.65 |  |  |  | 8.70 | 1.22 | 19.62 |  |  |
| ≥85 years old | Omicron |  | 8.42 | -14.57 | 37.75 | 21.56 | 0.002 |  | 8.12 | -14.67 | 38.03 | 21.34 | 0.002 |
|  | Non-Omicron |  | 15.44 | -11.52 | 48.81 |  |  |  | 15.50 | -11.49 | 47.96 |  |  |
| Male | Omicron |  | 0.97 | -0.13 | 2.55 | 19.78 | <0.001 |  | 0.96 | -0.13 | 2.55 | 20.04 | <0.001 |
|  | Non-Omicron |  | 1.62 | 0.20 | 4.35 |  |  |  | 1.62 | 0.20 | 4.29 |  |  |
| Female | Omicron |  | 0.78 | -0.43 | 2.32 | 20.15 | <0.001 |  | 0.77 | -0.44 | 2.31 | 20.42 | <0.001 |
|  | Non-Omicron |  | 1.39 | -0.18 | 3.70 |  |  |  | 1.39 | -0.18 | 3.68 |  |  |

**Table S3.** Wilcoxon test for weekly excess mortality when using a two-week average to fill the missing value of the variants.

|  | Variants |  | Median | P_25_ | P_75_ | *Z* | *P* |  |
| --- | --- | --- | --- | --- | --- | --- | --- | --- |
| All | Omicron |  | 0.85 | -0.22 | 2.22 | 19.91 | <0.001 |  |
|  | Non-Omicron |  | 1.47 | 0.05 | 4.01 |  |  |  |
| 0-14 years | Omicron |  | -0.02 | -0.15 | 0.11 | 23.28 | 0.662 |  |
|  | Non-Omicron |  | -0.02 | -0.15 | 0.11 |  |  |  |
| 15-64 years old | Omicron |  | 0.20 | -0.05 | 0.52 | 18.49 | <0.001 |  |
|  | Non-Omicron |  | 0.39 | 0.07 | 1.14 |  |  |  |
| 65-74 years old | Omicron |  | 1.18 | -1.37 | 4.41 | 18.18 | <0.001 |  |
|  | Non-Omicron |  | 2.95 | 0.17 | 8.75 |  |  |  |
| 75-84 years old | Omicron |  | 7.42 | 0.38 | 15.11 | 21.15 | <0.001 |  |
|  | Non-Omicron |  | 8.71 | 1.20 | 19.74 |  |  |  |
| ≥85 years old | Omicron |  | 8.20 | -14.42 | 37.66 | 21.59 | 0.002 |  |
|  | Non-Omicron |  | 15.44 | -11.53 | 48.83 |  |  |  |
| Male | Omicron |  | 0.76 | -0.44 | 2.30 | 19.82 | <0.001 |  |
|  | Non-Omicron |  | 1.40 | -0.17 | 3.73 |  |  |  |
| Female | Omicron |  | 0.97 | -0.12 | 2.55 | 20.14 | <0.001 |  |
|  | Non-Omicron |  | 1.62 | 0.20 | 4.35 |  |  |  |
